# Effect of Socialization on Alzheimer’s Disease During the COVID-19 Pandemic

**DOI:** 10.7759/cureus.66942

**Published:** 2024-08-15

**Authors:** Ricardo Maldonado-Puebla, Parth M Raina, Deepesh Khanna

**Affiliations:** 1 Medicine, Dr. Kiran C. Patel College of Osteopathic Medicine, Nova Southeastern University, Fort Lauderdale, USA; 2 Medicine, Dr. Kiran C. Patel College of Osteopathic Medicine, Nova Southeastern University, Clearwater, USA; 3 Foundational Sciences, Dr. Kiran C. Patel College of Osteopathic Medicine, Nova Southeastern University, Clearwater, USA

**Keywords:** social support intervention, social network, dementia, corona virus, neurodegenrative disease, covid-19, social engagement, social-isolation, alzheimer’s dementia, alzheimer’s diseases

## Abstract

Age is the strongest risk factor for Alzheimer’s disease, a neurodegenerative disease where beta-amyloid plaques accumulate in the brain. Elderly individuals, especially those in nursing homes, were burdened by social isolation during the COVID-19 pandemic. The purpose of this literature review is to describe the effectiveness of social engagement and how combating isolation can have a neuroprotective effect on individuals at risk for Alzheimer’s disease. We conducted a search in PubMed examining articles from 2010 to 2023 that discussed the impact of socialization on Alzheimer’s disease, particularly during the COVID-19 pandemic. Our search terms were “Alzheimer’s Disease + Socialization," “Social Isolation + Alzheimer’s Disease," “Alzheimer’s Disease + COVID-19," “COVID-19 + Social Isolation," and “Social Interventions + Alzheimer’s Disease." Inclusion criteria consisted of patients ages 60 and older with Alzheimer’s disease, mention of social isolation or engagement, and any relationship between COVID-19 and Alzheimer’s disease. Exclusion criteria were defined as other dementias, non-social interventions, and the effects of different viruses on Alzheimer’s disease. After the screening process, 30 articles were included, along with six articles that were suitable to the topic. Of the 36 total articles, 19 focused on an intervention involving socialization; eight explored the effect of social isolation during COVID-19 on patients with Alzheimer’s disease; five articles examined social isolation as a risk factor for dementia; and four articles discussed the effect of socialization on Alzheimer’s disease. A few studies reported that having a large social network can improve cognition and mood for patients with Alzheimer’s disease. Studies reported that interventions such as volunteering, video calls, group art classes, animal interactions, and others produced positive outcomes in Alzheimer’s patients, but not all were statistically significant. Our review found a consistent association between a socially integrated lifestyle and a decreased incidence of early-onset dementia. Although not all interventions were solely social, a strong social structure remained at the core of a healthy aging process.

## Introduction and background

The elderly population, which is defined as ages 65 and older, was impacted heavily by the COVID-19 pandemic [1-4]. This was evident in nursing homes, where elderly patients' weakened immune systems put them at great risk of contracting the virus. Due to the mandatory quarantine period after contracting COVID-19, residents were separated even further from their loved ones as a protective measure for their health. Within the homes, residents struggled with maintaining long-lasting relationships with fellow residents, as one in 10 nursing home residents died from COVID-19 in 2021 [5]. Social isolation and age are significant risk factors for Alzheimer’s disease, and it is estimated that currently, 6.5 million Americans aged 65 and older are living with Alzheimer’s disease [6]. Furthermore, the United States is becoming an aging nation, as it is estimated that by 2060, nearly one in four Americans will be 65 years and older [7]. With the social isolation brought on by the pandemic and the growing elderly population, the increased susceptibility to Alzheimer's disease soon is certainly cause for concern. 

Alzheimer’s disease is the most common form of dementia [8]. Dementia is a disorder that is characterized by impairment of cognition, typically involving memory, and at least one other cognitive impairment [9]. Alzheimer’s disease is a neurodegenerative disease where extracellular beta-amyloid plaques and intracellular neurofibrillary tangles accumulate in the brain, interfering with the brain’s neuronal circuits [9]. The symptoms of Alzheimer's disease include memory loss, difficulty speaking, difficulty completing tasks, increased confusion, etc. Currently, acetylcholinesterase inhibitors are used to treat the symptoms of Alzheimer’s disease, but there is no cure for Alzheimer’s disease, demonstrating the need for any interventions that can be of use [10].

While continuous efforts are being made towards cognitive behavioral therapy and pharmacological therapeutics in addressing the disease progression of Alzheimer’s, social engagement remains one of the most effective non-pharmacological interventions. Socialization is a lifelong process that not only is attributed to positive mental health but also a foundation for developing lifelong core personality traits and sharpening one’s emotional awareness and overall cognitive function. A growing number of studies have focused on isolating the effects of socialization on decreasing the risk of cognitive or memory impairment as seen in Alzheimer’s disease patients. While five- and 10-year longitudinal studies have shown mixed results regarding significant changes in cognitive function, results have consistently shown a strong positive effect on the overall quality of living for Alzheimer’s disease patients, as demonstrated by decreased risks for clinical depression and overall improvement in attention and memory. As important as it is to strengthen social relationships for patients affected by symptoms of early-stage dementia, the extent of benefits is limited without also changing other lifestyle factors. As a result, physicians continue to employ comprehensive treatment plans that not only involve encouraging the patient to maintain a positive social environment but also enforce long-term changes in dietary habits, diverse physical activity, and necessary pharmacological interventions that may enhance cognitive function and memory retention [11].

The primary objective of our study is to determine if socialization can be utilized as an effective intervention for those impacted by Alzheimer’s disease, with special consideration for those affected by social isolation during the COVID-19 pandemic. Our secondary objective is to research whether social isolation can be a risk factor for the progression of Alzheimer’s disease.

## Review

The study design is a literature review with a focus on examining the effect of socialization on Alzheimer’s disease. Specifically, our primary objective was to determine how socialization affected the pathophysiology and overall progression of Alzheimer’s disease with a keen intent on social isolation. The consequences of social isolation were most glaring during the COVID-19 pandemic when the vast majority of the senior community was quarantined for a prolonged period with minimal social engagement. Our secondary objective was to discover the effects of different socialization interventions on the development of dementia and Alzheimer’s disease in comparison to other treatment modalities.

**Figure 1:**
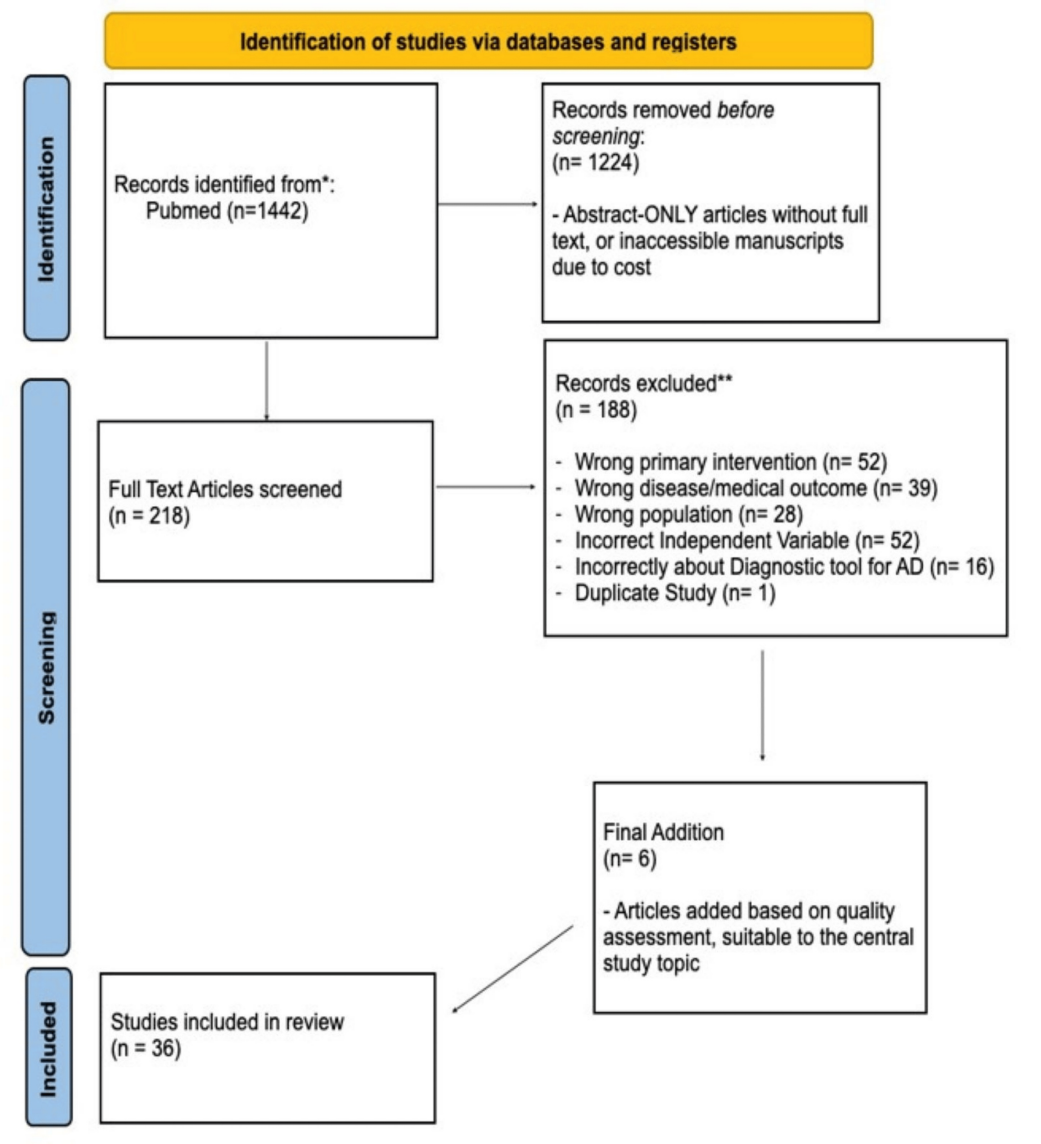
A PRISMA flowchart outlining the study selection process *PubMed; records were identified based on the keywords used. **Records were excluded based on the inclusion and exclusion criteria. PRISMA: Preferred Reporting Items for Systematic Reviews and Meta-Analyses

To accomplish our objectives, we performed a database search in PubMed. We used the search terms “Alzheimer’s Disease + Socialization," “Social Isolation + Alzheimer’s Disease," “Alzheimer’s Disease + COVID-19," “COVID+19 + Social Isolation," and “Social Interventions + Alzheimer’s Disease” while also limiting the publication dates to 2010-2023. The initial search resulted in a total of 1,442 citations. Inclusion criteria were created to screen the results, which excluded abstracts that had no associated full text, manuscripts that were not accessible due to cost, and books and documents. Following this preliminary screening, the initial exclusion criteria removed 1,224 articles, leaving 218 articles still eligible. Then further final criteria were created, and that consisted of patients ages 60 and older who were diagnosed with Alzheimer’s disease, some form of either social isolation or social engagement, and any relationship between COVID-19 and Alzheimer’s disease. Exclusion criteria consisted of other dementias such as frontotemporal or vascular dementia, interventions that were not socially based, the effects of other viruses on social isolation, or Alzheimer’s disease. This final inclusion and exclusion criteria narrowed our window down to 30 articles, as it most importantly removed any of the articles that had an undue emphasis on interventions that were not emphatically tied to social interventions. Of the 218 articles, 188 articles were excluded in our final screening process, among which 52 were excluded due to a wrong intervention (i.e., exercise, music therapy), 39 were excluded due to a wrong outcome/disease studied (i.e., stroke, bipolar disorder), 28 were excluded due to the wrong population (i.e., caregivers, children), 52 were excluded due to an incorrect independent variable with Alzheimer’s disease (i.e., genetics, particulate matter), 16 were excluded due to the study being about a diagnostic tool of Alzheimer’s disease, and one was excluded as it was a duplicated study. In addition, six articles were included independent of the literature search as they were suitable to the topic of the paper. Figure 1 shows the study selection process. 

Results

Table 1 summarizes the main findings of the studies included in the review. 

**Table 1:** Main findings of the studies included in the review showing the correlation between dementia risk and social activities/isolation

Author	Study design	Title	Main findings
Marioni et al., 2015 [12]	Cohort study	Social activity, cognitive decline, and dementia risk: a 20-year prospective cohort study	A medium and high level of social engagement both significantly reduced the rate of cognitive decline and overall dementia risk. A strong sense of self-perception led to decreased rates of cognitive decline in the long term.
Almeida-Meza et al., 2021 [13]	Longitudinal study	Is Engagement in Intellectual and Social Leisure Activities Protective Against Dementia Risk? Evidence from the English Longitudinal Study of Ageing	It showed a strong negative association between dementia risk and social/intellectual leisure activities but was only statistically significant for individuals who were married as opposed to single or widowed. Specific leisure activities such as reading the newspaper for females, mobile phone usage in males, and hobbies for married individuals were significant in lowering the risk of dementia.
Shen et al., 2022 [14]	Cohort study	Associations of Social Isolation and Loneliness With Later Dementia	Social isolation was associated with a 26% increased likelihood of dementia. Structural differences were noted from MRI analyses in socially isolated patients showing reduced gray matter volumes in the hippocampal region and increased incidence of mitochondrial dysfunction and oxidative stress.
Popa et al., 2021 [15]	Clinical trial	Impact of Alzheimer’s Dementia on Caregivers and Quality Improvement through Art and Music Therapy	Studies reported that music and art therapy can increase socialization and group membership in Alzheimer’s patients and that it can decrease social isolation. Music therapy has been shown to reduce anxiety, depression, and cortisol levels in patients with dementia, while art therapy can increase cognitive and motor functioning.
Kuo et al., 2020 [16]	Review	Association of Late Life Depression, (Non-) Modifiable Risk and Protective Factors with Dementia and Alzheimer's Disease: Literature Review on Current Evidences, Preventive Interventions and Possible Future Trends in Prevention and Treatment of Dementia	Reported that individuals who have strong social engagement with family and friends are less likely to develop dementia later in life. Positive social support can reduce stress, a risk factor for Alzheimer’s disease. Socialization can stimulate cognitive ability and benefit the immune system by preserving the cellular stability of the brain.
Lebrasseur et al., 2021 [17]	Review	Impact of the COVID-19 Pandemic on Older Adults: Rapid Review	Isolation during the COVID-19 pandemic may exacerbate issues in the elderly such as loneliness, anxiety, and age discrimination. Determined that older individuals reported a worsening of psychological symptoms and greater loneliness during the pandemic.
Manca et al., 2020 [18]	Review	The Impact of COVID-19 Infection and Enforced Prolonged Social Isolation on Neuropsychiatric Symptoms in Older Adults With and Without Dementia: A Review	Alzheimer’s patients hospitalized with COVID-19 presented with higher rates of agitation and delirium. Alzheimer’s patients not infected with COVID-19 but who were socially isolated reported a worsening of their neuropsychiatric symptoms. Patients with Alzheimer’s disease who had larger social networks were able to have better cognitive performance.
Hsiao et al., 2018 [19]	Mechanistic study	Impact of social relationships on Alzheimer's memory impairment: mechanistic studies	It is inclusive to state that social isolation is a risk factor for Alzheimer’s disease, as evidence differs if the beta-amyloid plaques seen on brain autopsies are correlated with social isolation and loneliness.
Bellou et al., 2017 [20]	Meta-analysis	Systematic evaluation of the associations between environmental risk factors and dementia: An umbrella review of systematic reviews and meta-analyses	Found a statistically significant association between the low frequency of social contact and the development of all types of dementia, including Alzheimer’s disease. In addition, they stated that benzodiazepine use, depression, late-life depression, and type 2 diabetes are associated with all types of dementia.
Duan et al., 2018 [21]	Meta-analysis	Psychosocial interventions for Alzheimer's disease cognitive symptoms: a Bayesian network meta-analysis	Reported that psychosocial interventions are effective in improving cognitive function in Alzheimer’s disease patients. Demonstrated that cognitive effects were better for group exercise, music therapy, walking programs, and reminiscence therapy than usual treatment with acetylcholinesterase inhibitors.
Ortiz et al., 2022 [22]	Review	Alzheimer’s Disease and SARS-CoV-2: Pathophysiological Analysis and Social Context	Suggested a synergistic relationship between COVID-19 and Alzheimer’s disease as both are associated with increased levels of inflammatory markers, APOE-4ε, ACE2, and oxidative stress that are involved in cognitive impairment. Forty patients with Alzheimer’s disease during five weeks of confinement during the COVID-19 pandemic experienced increased levels of agitation, apathy, and impaired motor activity. Video calls from loved ones resulted in higher levels of satisfaction.
Simonetti et al., 2020 [23]	Review	Neuropsychiatric Symptoms in Elderly With Dementia During COVID-19 Pandemic: Definition, Treatment, and Future Directions	Found that apathy was the most reported symptom to manifest from social isolation during the pandemic for Alzheimer’s patients. Reported that the implementation of electronic services of physical exercise, sensory stimulation, reminiscence-based brain health, music therapy, and other creative activities for people with dementia did not significantly improve their behavior.
Lam et al., 2021 [24]	Systematic review	Neurobiology of loneliness: a systematic review	It was found that increases in loneliness resulted in increased white matter hyperintensities in older individuals without dementia. It indicated a significant relationship between loneliness and increased amyloid burden and increased tau pathology in the right entorhinal cortex and right fusiform gyrus, suggesting a heightened risk of developing Alzheimer’s disease.
Morgan- Gouveia et al., 2022 [25]	Review	Geriatric update 2022: Preventing Alzheimer's disease and more	Reported that for 1,427 older adults in the Netherlands, frailty was associated with increased mortality risk and that frailty in combination with low social support or loneliness increased mortality. It stated that the six-month mortality rate for patients with COVID-19 and dementia was 21% which was higher compared to the 4.8% without dementia.
Tragantzopoulou et al., 2021 [26]	Review	Social isolation and loneliness in old age: Exploring their role in mental and physical health	Reported that satisfactory social interactions are protective against dementia for over 15 years. The size and type of the social network a person has is not protective against dementia. They ultimately stated that the relationship between social isolation and the onset of Alzheimer’s disease is still relatively unknown.
Gaigher et al., 2022 [27]	Systematic review	Dementia and Mental Health During the COVID-19 Pandemic: A Systematic Review	Found that the symptoms that increased the most for 40 patients with Alzheimer’s disease were apathy, agitation, and aberrant motor behavior which was different than for those with mild cognitive impairment (40 patients) during social isolation in the pandemic. Determined that video calls and phone calls can be a source of social support but that these interventions were not statistically significant for changing health and well-being in 93 patients with mild dementia or mild cognitive impairment.
Sindi et al., 2015 [28]	Systematic review	Advances in the Prevention of Alzheimer’s Disease	Reported that socially stimulating factors, exercise, and high levels of education are protective factors against Alzheimer’s disease and dementia. For 1260 community-dwelling adults at risk for dementia, a multimodal intervention that consisted of nutritional guidance, exercise, cognitive training, social activity, and intensive monitoring of metabolic/vascular risk factors had improved Neuropsychological Test Battery scores by 25% compared to the control group.
Kim et al., 2021 [29]	Review	Neurological Mechanisms of Animal-Assisted Intervention in Alzheimer's Disease: A Hypothetical Review	Found that in 10 patients with Alzheimer’s disease, social interaction with animals can decrease loneliness, anxiety, fear, and sadness for patients with Alzheimer’s disease. Discovered that for 20 patients with Alzheimer’s disease who had interaction with an animal during therapy, their Geriatric Depression Scale and Mini Mental Status Exam scores improved. Tau hyperphosphorylation and amyloid-beta (Aβ) pathology are exacerbated by social isolation.
Gaugler et al., 2019 [30]	Introductory article	Reconsidering frameworks of Alzheimer's dementia when assessing psychosocial outcomes	Determined that person-centered care focused on a positive social environment, valuing the patient, treating them as individuals, and considering their perspective should be implemented as it is relatively good for Alzheimer’s patients’ well-being.
Eubank et al., 2022 [31]	Review	Effects of Lifestyle Factors on Cognition in Minority Population of Older Adults: A Review	Reported that for 617 African American older adults, social engagement improved cognitive function as social interaction helped reduce stress and depression. Stated that the negative effects of social isolation are worse for those with low income or of color, especially if they are elderly individuals alone in a neighborhood.
Yee et al., 2018 [32]	Review	Alzheimer’s disease: insights for risk evaluation and prevention in the Chinese population and the need for a comprehensive program in Hong Kong/China	Stated that having an occupation and participating in leisure activities can reduce the risk of developing dementia. Described that multimodal interventions should include cultural appropriateness, such as for Chinese patients can correspond treatment with Confucian Six Arts where social functioning is one of the domains.
Sadowsky et al., 2012 [33]	Review	Guidelines for the management of cognitive and behavioral problems in dementia	Reported that non-pharmacological interventions such as social support should always be used in conjunction with pharmacological therapy. The way you communicate with Alzheimer’s patients is significant, and people should utilize the three R’s approach: repeating, reassuring, and redirecting, which can reduce agitation and anxiety in patients with dementia.
Delfa-Lobato et al., 2021 [34]	Systematic review	Benefits of Cultural Activities on People With Cognitive Impairment: A Systematic Review	Storytelling and visual arts were two cultural interventions that enhanced socialization in individuals with dementia. Cultural interventions can improve general cognition, quality of life, emotional well-being, self-esteem, and communication for participants with cognitive impairment but are not significant enough for memory, language, and daily functioning.
Huntley et al., 2015 [35]	Meta-analysis	Do cognitive interventions improve general cognition in dementia? A meta-analysis and meta-regression	Found that cognitive stimulation that included social activities improved the scores on the Mini Mental Status Exam and Alzheimer's Disease Assessment Scale-Cognition for those with dementia, but this was not clinically significant.
Nunez da Cruz Morello et al., 2017 [36]	Systematic review	Language and communication non-pharmacological interventions in patients with Alzheimer's disease: a systematic review. Communication intervention in Alzheimer	Reported that for 42 individuals with Alzheimer’s disease with an intervention that included exercise and language stimulation sessions along with social and volunteer experiences, they had stable cognitive function, mood, and fitness. Lexical semantic interventions demonstrated the most benefit on communication skills for Alzheimer’s patients, but interventions based solely on conversation need more research.
Bae et al., 2019 [37]	Randomized controlled trial	The effect of a multicomponent intervention to promote community activity on cognitive function in older adults with mild cognitive impairment: A randomized controlled trial	Reported that for 83 individuals with mild cognitive impairment, social activity, physical activity, and cognitive sessions had a significant effect on their spatial working memory. However, Mini Mental Status exams and composite word memory showed no difference between the interventional group and the control group.
Kumar et al., 2021 [38]	Review	The Long-Term Public Health Impact of Social Distancing on Brain Health: Topical Review	Found that individuals with a genetic risk for Alzheimer’s disease may be at greater risk of loneliness and social isolation. Individuals with a smaller social network have been found to have smaller left posterior superior temporal sulci, middle temporal gyri, and entorhinal cortices.
Plasman et al., 2010 [39]	Systematic review	Systematic review: factors associated with risk for and possible prevention of cognitive decline in later life	Reported no consistent association between social networks, social support, marital status, and cognitive decline in older adults. Lifestyle interventions need to be done as early as possible in Alzheimer’s patients to demonstrate an effect.
Anstey et al., 2013 [40]	Randomized controlled trial	A 12-week multidomain intervention versus active control to reduce risk of Alzheimer's disease: study protocol for a randomized controlled trial	For 12 weeks, 180 participants at risk for Alzheimer’s disease, utilized an intervention that consisted of seven online modules: dementia literacy, risk factor education, engagement in physical, social, and cognitive lifestyles, nutrition, and health monitoring; however, nothing conclusive has been stated from their intervention. Stated that there are no interventions that reduce the risk of Alzheimer’s disease delivered over the internet, including social ones.
Droes et al., 2019 [41]	Randomized controlled trial	Utilization, effect, and benefit of the Individualized Meeting Centers Support Program for people with dementia and caregivers	Found that having people with dementia work as volunteers in society by using their talents was able to improve their neuropsychiatric symptoms and affect. Found that when compared to having no social support, being able to volunteer in society or doing telephone coaching made the individuals happier.
Charlesworth et al., 2016 [42]	Randomized controlled trial	Peer support and reminiscence therapy for people with dementia and their family carers: a factorial pragmatic randomized trial	Reported that peer support versus no peer support resulted in a 0.52 increase in the health-related quality of life test, the SF-12. Overall, determined that there is no evidence that peer support or reminiscing therapy can improve the quality of life for those with dementia.
Chiu-Wa Lam et al., 2015 [43]	Randomized controlled trial	Would Older Adults with Mild Cognitive Impairment Adhere to and Benefit from a Structured Lifestyle Activity Intervention to Enhance Cognition?: A Cluster Randomized Controlled Trial	Found a decrease in subjective cognitive complaints and depressive symptoms for the intervention groups that were physical exercise, cognitive activity, integrated cognitive and physical exercise, and social activity for 555 adults with mild cognitive impairment. Reported that these structured lifestyle interventions were not associated with changes in everyday functioning.
Dannhauser et al., 2014 [44]	Randomized controlled trial	A complex multimodal activity intervention to reduce the risk of dementia in mild cognitive impairment--ThinkingFit: pilot and feasibility study for a randomized controlled trial	Found that for 67 individuals with mild cognitive impairments, an intervention that consisted of physical activity, group cognitive stimulation, which included social interaction, and individual cognitive stimulation helped decrease BMI and systolic blood pressure and improved cognition.
Salinas et al., 2017 [45]	Cohort study	Associations between social relationship measures, serum brain-derived neurotrophic factor, and risk of stroke and dementia	Found that social connectedness was not associated with increased brain-derived neurotrophic factor (BDNF). Having a source of constant emotional support was associated with an increased level of BDNF. In addition, having someone to listen to and talk to was associated with a decreased incidence of stroke and dementia.
Murroni et al., 2021 [46]	Systematic review	Effectiveness of Therapeutic Gardens for People with Dementia: A Systematic Review	Found that having an outdoor community garden for individuals with Alzheimer’s can promote creativity, self-esteem, social interaction, sensory stimulation, gross and fine motor skills, and hand-eye coordination.
Lara et al., 2020 [47]	Randomized controlled trial	Neuropsychiatric Symptoms and quality of life in Spanish patients with Alzheimer's disease during the COVID-19 lockdown	Reported that neuropsychiatric symptoms worsened during the pandemic due to social isolation and that the symptoms most affected were agitation, apathy, and abnormal motor activity.

Discussion

In our review, we explored the relationship between socialization and Alzheimer’s disease, and what impact it has on the progression of the disease. We also considered the effect of social isolation as a risk factor for dementia and Alzheimer’s disease. Our extensive search of articles supplied several key findings. A major fundamental finding was that several social interventions played a role in improving cognition, mood, stress, anxiety, depression, and other symptoms of Alzheimer’s disease. However, not all social interventions were statistically significant. Another major key finding was that social isolation is harmful for patients with Alzheimer’s disease, as demonstrated by multiple randomized controlled trials during the COVID-19 pandemic. Social isolation was also associated with anatomical changes in the brains of patients with Alzheimer’s disease. The evidence states that socialization may be neuroprotective, but there is not sufficient research data to prove it. However, socialization should still be considered for patients at risk of Alzheimer’s disease and for those currently diagnosed with Alzheimer’s disease as the benefit supersedes any risk involved.

Effect of Social Isolation and Social Engagement on Alzheimer’s Disease

Our systematic review investigated the underlying associations between social activity and the progression of dementia. Supported by an overwhelming collection of evidence, we found a consistent correlation between social isolation in both human and animal populations and the resulting exacerbation of Alzheimer’s disease. The debilitating effects of end-stage Alzheimer’s disease were directly observable by significant reductions in cognitive abilities and short- and long-term memory retention [12, 13]. The effects of social isolation and loneliness on late-stage Alzheimer’s were strongly compounded by the COVID-19 pandemic that forced seniors across the globe to remain quarantined for several months if not years. Seniors were either confined to nursing homes or stuck with a single caregiver in a remote setting, which made it difficult to incorporate dynamic environmental changes in their lives. But even outside of the pandemic, static social activity has long been a public health concern for older seniors who may have recently lost a spouse, have limited contact with their children and grandchildren, or are presented with obstacles that hinder their ability to make connections within their community. The ramifications of social isolation have been documented in longitudinal studies through investigations of underlying neuropathology. Demonstrated in both human and animal study cohorts, long-term social isolation was accompanied by decreased hippocampal volume, which is integral to the retention and formation of new memories, and decreased prefrontal cortex volume, essential to higher-order cognitive abilities [19]. Subjects of these studies also demonstrated decreased dendritic spine density and neuronal connections, largely a consequence of amyloid-beta plaques that accelerate the progression of late-stage dementia to Alzheimer’s disease [19].

In contrast to the premise of social isolation presenting as a strong risk factor for Alzheimer’s deterioration, increased social engagement has been shown to strongly improve general prognosis and even delay the onset of dementia or Alzheimer’s disease for individuals who may have a greater genetic disposition [14]. Studies have suggested that any type of physical or social activity serves as a protective effect against a variety of neurodegenerative disorders [48]. Specifically in the case of patients with Alzheimer’s and late-stage dementia, engaging in a wide variety of hobbies, interacting with a spouse or a loved one regularly, or participating in intellectually demanding tasks all have strongly positive effects in that they limit the debilitating symptoms of Alzheimer’s and preserve core cognitive abilities and emotional intelligence. Results are mixed and ill-defined as to the certain threshold of social engagement or networking that yields conclusive results in decreased incidence of dementia [49]. In addition, there is ambiguity regarding the characterization of certain activities like internet or mobile phone usage as mentally stimulating tasks or not, and whether short vs. long-term usage leads to significant changes in cognitive abilities in Alzheimer’s disease patients compared to baseline. It is important to put into context that many studies evaluate variables that have significant overlap, including social, physical, and environmental factors that make it hard to discern the relatively isolated impact of each of those factors on long-term Alzheimer’s progression. However, it is clear that all of these variables exhibit a strong negative correlation to cognitive decline and that patients who are most likely to thrive and remain immune to the debilitating symptomatology of Alzheimer’s disease are the ones remaining active through intellectually demanding leisure activities with companions [49]. As most past and present research studies have analyzed Alzheimer’s disease through the spectrum of cognitive decline, future research needs to address the other physiological and functional deficits noted in Alzheimer’s disease patients [14, 48, 49]. It will be critical to objectively compare the impact of social isolation or, conversely, engagement on significant decline in anterograde and retrograde memory or behavioral and core personality changes, all of which are known to be demonstrably affected in end-stage Alzheimer’s disease.

Social Isolation During COVID-19 Increases the Risk of Alzheimer’s Disease

The COVID-19 pandemic has had an unprecedented impact on the world, affecting the health of billions. Countries took several measures to combat the spread of COVID-19, one being quarantining, which led many to live a life of social isolation, further affecting the physical and mental health of others [50-55]. Social isolation is defined as the experience of having few purposeful social relationships and having minimal social contact with others. Social isolation increased significantly during the pandemic; as per a multinational survey (101 different countries), a fifth of people (21%) defined themselves as socially isolated based on their usual relationships. In addition, 13% of people reported that they experienced a substantial increase in social isolation than the social isolation they had before the pandemic [56]. Social isolation has been recognized as a risk factor for morbidity and mortality, as studies have shown it to be associated with increased cardiovascular disease, cognitive deterioration, and infectious illness [57]. The World Health Organization declared COVID-19 a pandemic in March 2020. In 2019, an estimated 5.8 million people were living with Alzheimer’s disease. In 2021, that number increased to 6.2 million. As of today, 6.5 million Americans 65 and older are living with Alzheimer’s disease. By 2050, the number of people aged 65 and older with Alzheimer's disease is estimated to increase significantly to 12.7 million, with the COVID-19 pandemic being one of the main catalysts for that large increase [58].

Furthermore, social isolation during the COVID-19 pandemic affected those already diagnosed with Alzheimer’s disease, as it worsened many of the symptoms they experience daily. Lara et al. (2020) found that neuropsychiatric symptoms worsened during the pandemic due to social isolation and that the symptoms most affected were agitation, apathy, and aberrant motor activity [47]. As symptoms worsened, deaths due to Alzheimer’s disease rose significantly during the pandemic, as the COVID-19 pandemic caused Alzheimer’s deaths to increase by approximately 16% more than expected [6]. In addition, mortality rates for those with dementia infected with COVID-19 were higher than those without dementia [25]. Certain symptoms that worsened were different for those with Alzheimer’s disease compared to those with mild cognitive impairment [27]. While symptoms worsened during the social isolation throughout the pandemic, certain social interventions were attempted, such as video and phone calls, but these were not statistically significant for two studies [23, 27] but were statistically significant for two other studies demonstrating conflicting evidence [18, 22].

Studies have demonstrated a bidirectional relationship between COVID-19 and the prognosis of Alzheimer’s disease. COVID-19 causes a rise in the same inflammatory markers that arise with Alzheimer’s disease [22]. While Alzheimer’s is synonymous largely with cognitive instability and memory lapses, a significant percentage of Alzheimer’s disease patients report chronic suffering from depression and anxiety symptoms. A large ramification of the COVID-19 pandemic, especially in the early onset with no prevalent vaccination or efficacious therapeutics, was lengthy lockdown periods that had detrimental consequences on a vast majority of individuals. There were immediate lifestyle changes that needed to be adopted for the safety of elderly individuals, yet at the risk of social isolation and lack of physical activity, this vulnerable population of individuals with various neurocognitive disorders was increasingly susceptible to loneliness and depression. Large-scale quarantines led to a decrease in overall quality of living as most countries and cities were initially ill-prepared to deal with the effects of a global pandemic, especially for those living in nursing homes or even those with no caregiver at home. While investigations and further research continue into the long-term consequences of COVID-19, increasingly prevalent analysis shows the critical nature of social engagement in impeding the prognosis of Alzheimer’s disease and other neurodegenerative disorders.

Social Interventions Improve the Symptoms of Alzheimer’s Disease

Alzheimer’s disease is a brain disorder that progressively destroys nerve cells, resulting in memory loss and behavioral impairment. The cardinal symptom of Alzheimer’s disease is memory impairment; however, there are other symptoms that those with Alzheimer’s disease can experience, such as expressive aphasia, behavioral apathy, irritability, executive dysfunction, apraxias, sleep disturbances, and seizures. Alzheimer’s is a disease that typically affects people later in life, and the most important risk factor for Alzheimer’s disease is age. After reaching 65 years of age, the risk of Alzheimer’s disease rises exponentially [59]. Therefore, it is important to intervene early in a person’s life to slow down the progression towards Alzheimer’s disease and mitigate the controllable risk factors such as high cholesterol and high blood pressure [60]. Social interventions that occur early in life for those at risk can be one of the simple ways to reduce the likelihood of Alzheimer’s disease developing at an older age.

Several studies have implemented different types of social interventions and observed the impact of those interventions on Alzheimer’s patients [29,45,61]. An important intervention for those diagnosed with Alzheimer’s disease is to make sure they are involved in a community. Several interventions were implemented across several studies, including volunteering in the community, coaching, group exercises, group music therapy, art therapy, reminiscence therapy, group cognitive stimulation, storytelling, visual arts, and cultural therapy, and these helped improve neurological function for those with Alzheimer’s disease [39-42]. Results were collected from the Mini-Mental Status Exams, Geriatric Depression Scales, Neuropsychological Test Battery, Alzheimer’s Disease Assessment Cognition Scale, 12-item Short Form Survey (SF-12), and others to measure increases in cognitive function. However, not all socialization interventions produced statistically significant increases in cognition. Further research needs to be done to determine which type of social intervention is superior so that it can be promoted more.

Another social intervention that is beneficial in decreasing memory impairment is having a source of constant emotional support. In a study by Salinas et al. (2017), they researched the association between socialization and brain-derived neurotrophic factor (BDNF) in humans with Alzheimer’s disease from the Framingham Heart Study cohort. Socialization was broken down into two main components: social connectedness and emotional support [45]. They found that social connectedness was not associated with increased BDNF; however, having a source of constant emotional support was associated with an increased level of BDNF. In addition, having someone to listen to and talk to was associated with a decreased incidence of stroke and dementia [45]. Examples of where patients can obtain this specific type of support are from their families, a psychologist, friends, and support groups.

An additional social intervention that can assist Alzheimer’s patients with some of their related symptoms is animal-assisted. Having an animal companion has been shown to reduce loneliness, anxiety, and fear in Alzheimer’s patients while also improving cognition. Sundown syndrome, a symptom of Alzheimer’s disease, is a phenomenon that describes the transition of Alzheimer’s patients becoming more restless, confused and agitated as the day progresses. It was found that therapy dogs increased socialization for Alzheimer’s patients, as a change in the patient’s body language was observed as they communicated verbally with the dog. In addition, they observed that the use of therapy dogs decreased agitation, especially during the sundown period for Alzheimer’s patients. This was regardless if the patient had high or low-level dementia, as very few agitated behaviors were observed by researchers during the study [29, 61]. In addition, a therapy dog can also remove some burdens from the caregiver of each patient. The removal of burdens such as fatigue, isolation, and anxiety can allow the caregiver to spend more time providing emotional support to the Alzheimer’s patient. However, the use of an animal-assisted intervention does not alleviate Alzheimer’s pathology, as research has only been done on symptoms of Alzheimer’s disease.

Sleep disturbances, changes in mood, and additional symptoms of Alzheimer’s disease can be eliminated by an outdoor community garden. Sleep disturbances occur in Alzheimer’s disease because, as dementia alters the brain cells, it simultaneously affects the circadian rhythm. Murroni et al. (2021) determined that behavior was the domain most improved by an outdoor community garden as it reduced aggressive behaviors, agitation, depression, and stress [46]. There is a difference between an outdoor garden and an indoor garden, as an outdoor community garden is better than an indoor community garden for improving behavior, cognition, mood, sleep, and the risk of falls. In regards to sleep, they found that having an outdoor community garden for individuals with Alzheimer’s can improve their sleep by allowing the patients to get adequate sunlight, which in turn can improve their circadian rhythm. Furthermore, having a horticultural garden can further stimulate social interaction and creativity and improve gross and fine motor skills, especially for those dealing with apraxias [46, 62].

Though many social interventions can help improve symptoms of Alzheimer’s disease, there is no cure for Alzheimer’s disease. Cholinesterase inhibitors, such as donepezil, galantamine, and rivastigmine, are the primary medications used for Alzheimer's patients. These acetylcholinesterase inhibitors increase acetylcholine at the synaptic cleft of the nerve ending to enhance nerve cell communication. However, it is unclear whether these medications can improve an Alzheimer's patient's mental health [63]. This can be contrasted with social interventions, which have been shown in some studies to improve a patient's mental health. Not many studies in our review compared pharmacological therapy to social intervention, but in the study by Duan et al. (2018), they demonstrated that cognitive effects were better for group exercise, music therapy, reminisce therapy, and a walking program than with treatment with acetylcholinesterase inhibitors [21]. When individuals socialize, their brain produces more dopamine and lower cortisol levels, which is the hormone associated with stress levels in the body. Thus, socializing can help lower stress and aid in reducing depression and anxiety. There is a need for research on the combined effects of social interventions with pharmacological interventions and the comparison between the two.

## Conclusions

The central purpose of our systematic review was to critically assess the effect of social engagement, or the lack thereof, in patients with Alzheimer’s disease. In our analysis, we determined how social isolation acted as an independent risk factor in exacerbating the progressive neurodegenerative physiological changes noted in Alzheimer’s patients and, on the contrary, how maintaining social connections essentially bolstered cognitive function and working memory. An unfortunate effect of COVID-19 was exacerbating pre-existing public health crises of social isolation and loneliness. The pandemic was an exogenous force that created several impeding factors for a vulnerable senior population that subsequently correlated with a visible decline in overall cognitive function, along with worsening of depression and other neuropsychiatric symptoms. The next step in tackling early-onset dementia and Alzheimer’s disease involves finding effective social intervention strategies and combining these large-scale effects with further investigative research into other effective pharmaceutical agents that can not only manage symptoms but potentially reverse functional and cognitive decline in Alzheimer’s disease patients.
